# Harpy: a pipeline for processing haplotagging linked-read data

**DOI:** 10.1093/bioadv/vbaf133

**Published:** 2025-06-05

**Authors:** Pavel V Dimens, Ryan P Franckowiak, Azwad Iqbal, Jennifer K Grenier, Paul R Munn, Nina Overgaard Therkildsen

**Affiliations:** Department of Natural Resources and the Environment, Cornell University, Ithaca, NY 14853, United States; Department of Natural Resources and the Environment, Cornell University, Ithaca, NY 14853, United States; Department of Natural Resources and the Environment, Cornell University, Ithaca, NY 14853, United States; Genomics Innovation Hub, Biotechnology Resource Center, Cornell University, Ithaca, NY 14853, United States; Genomics Innovation Hub, Biotechnology Resource Center, Cornell University, Ithaca, NY 14853, United States; Department of Natural Resources and the Environment, Cornell University, Ithaca, NY 14853, United States

## Abstract

**Motivation:**

Haplotagging is a method for linked-read sequencing, which leverages the cost-effectiveness and throughput of short-read sequencing while retaining part of the long-range haplotype information captured by long-read sequencing. Despite its utility and advantages over similar methods, existing linked-read analytical pipelines are incompatible with haplotagging data.

**Results:**

We describe Harpy, a modular and user-friendly software pipeline for processing all stages of haplotagged linked-read data, from raw sequence data to phased genotypes and structural variant detection.

**Availability and implementation:**

https://github.com/pdimens/harpy.

## 1 Introduction

Next-generation sequencing has facilitated the rapid expansion of genetics and genomics in many different disciplines. Short-read sequencing technology continues to have unrivaled throughput and cost per base-pair, compared to “third-generation” long-read (full-molecule length) sequencing technologies such as those pioneered by Pacific Biosciences and Oxford Nanopore Technologies. The benefits of long-read sequencing include higher quality genome assemblies, longer phased haplotypes, and greater power and accuracy for identifying larger structural variants (Schrinner *et al.* 2020, Ahsan *et al.* 2023). Linked-read sequencing leverages the power of both technologies by using barcodes to tag fragments derived from the same molecule that can be tracked in the downstream data processing (Zheng *et al.* 2016). This maintains the cost-effectiveness, throughput, and quality of short-read technology while retaining part of the long-range information captured in third-generation long molecule sequencing. Typical short-read sequencing data, such as that provided by the Illumina platforms, is often insufficient to resolve distant variants into haplotypes, whereas linked-read data provides long-range information that can be leveraged to create these haplotype blocks in the attempt to reconstruct the full combination of alleles co-occurring on a parental chromosome. This technology has been used for genome assembly, population genomics, linkage mapping, cancer research, and other applications, but the major commercially available resources (from 10x Genomics) were discontinued in 2020. Since then, researchers have devised new methods such as TELL-seq (Chen *et al.* 2020), stLFR (Wang *et al.* 2019), and haplotagging (Meier *et al.* 2021). Among these, haplotagging is a non-commercial solution with improved cost per sample, reduced sample preparation complexity, and does not require proprietary instrumentation or custom sequencing configurations.

Access to user-friendly data processing and analysis pipelines facilitates the adoption of new sequencing approaches. For example, the availability of software suites like dDocent (Puritz *et al.* 2014) and STACKS (Catchen *et al.* 2011) played an important role in popularizing RAD-seq approaches. Similarly, the LongRanger software pipeline aided the adoption of the 10X Genomics linked-read technology but became abandoned in 2020 when the 10X Genomics linked-reads technology was discontinued. TELL-seq and stLFR are supported by bespoke software pipelines optimized for a fixed set of vendor-supplied barcodes, limiting the cross-platform use of these pipelines. Linked-read methods have already proven themselves to be very promising for population genomics studies, but broader adoption of haplotagging would likely be facilitated by end-to-end support from sample preparation to the initial data processing. To this end, we present Harpy, a user-friendly software pipeline that processes raw haplotagging data through demultiplexing, quality checks, alignment, variant calling, structural variant detection, imputing, and haplotype phasing, with additional workflows tailored to linked-read data.

## 2 Methods

**Figure 1.**
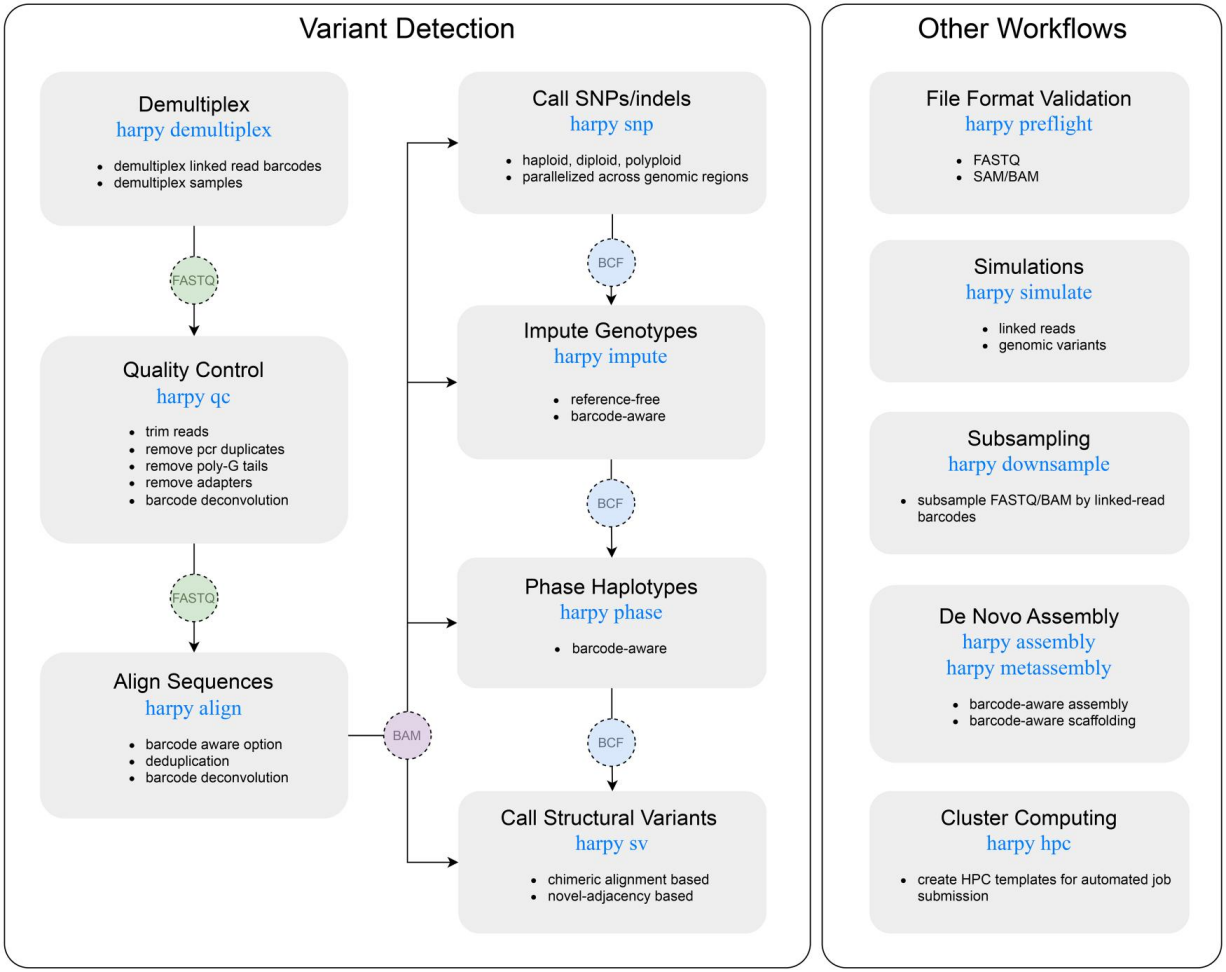
The data processing workflow of haplotagging linked-read data with Harpy modules. Subtitle text indicates the harpy command for the workflow, and the circles between boxes indicate the expected outputs and inputs for a given workflow.

**Figure 2.**
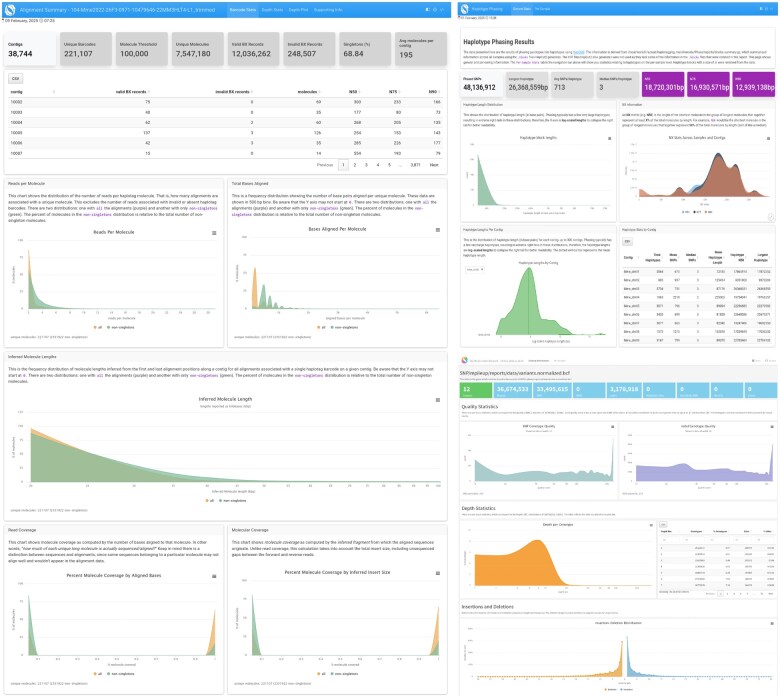
Interactive reports Harpy generates for alignment (left), haplotype phasing (right, top), and SNP calling (right, bottom) workflows.

Meier *et al.* (2021) introduced the haplotagging technique and provided their data processing and analysis methods in a public software repository (https://github.com/evolgenomics/HeliconiusHaplotagging) to complement their investigation. The Harpy built on top of those initial methods and was designed to be faster, more sophisticated, generalizable, modular (Fig. 1), parallelized, and easily distributed in a cluster-computing environment, with strong considerations for user adoption and experience. The different modules and routines are dispatched using the Snakemake workflow engine (Köster and Rahmann 2012), which was chosen for its utility, ubiquity, and legibility. Workflow engines execute code deterministically, where directed acyclic graphs resolve defined output targets based on defined inputs across interdependent tasks. This contrasts with scripting languages (e.g. R, Python, Julia), where code runs line-by-line from start to finish. Harpy uses Snakemake to automate input and output checking, container and environment creation, cluster computing submission, and incomplete workflow continuation. Scripting languages are more familiar to many users, often making the learning curve for deterministic languages like Snakemake notably steeper. The learning curve is then exacerbated by the dozens of Snakemake configuration options, irrespective of the parameterization of the underlying software the workflow will use for data processing. Thus, Harpy uses Snakemake internally as a pre-configured workflow engine, with each workflow exposed to the user as a simplified command-line program with clear options to set parameters for the most common or consequential arguments of the underlying software, with the ability to supply all other parameters as well. This abstraction also enables input file and parameter validation prior to Snakemake execution, which significantly aids in preventative troubleshooting for complex workflows. In addition to this infrastructure, Harpy generates interactive HTML reports with key metrics and visualizations for the relevant outputs of the different workflows (Fig. 2).

Harpy requires paired-end FASTQ-formatted files and a genome reference in FASTA format for the standard variant detection workflow. Subsequent workflows (e.g. phasing, SNP or SV detection, imputation) require BAM-formatted sequence alignment files and/or BCF- or VCF-formatted files of variants. The various modules of Harpy are designed to output properly formatted files for subsequent workflows. Alternatively, a data simulation workflow (to simulate genomic variants or raw linked-read sequencing data) requires at least one FASTA-formatted file as input.

## 3 Results

### 3.1 Demultiplexing

The demultiplexing routine processes raw Illumina haplotagging reads and transfers the barcode sequences into the FASTQ sequence header. Due to Harpy’s modular design, this workflow is extensible to additional demultiplexing strategies for modified or newly developed haplotagging barcoding methods. A typical barcoding format uses the BX: Z tag following the AxxCxxBxxDxx segment-aware format, where xx is an integer between 0–99 and 00 (e.g. B00) would indicate the barcode segment is invalid. The demultiplexing process uses a lookup table to associate a nucleotide barcode segment with its proper “ACBD” alias, removes the inline haplotagged barcode from the sequence, and then adds the ACBD barcode to the read header as e.g. BX: Z: A01C54B23D09. The BX: Z tag follows the proper SAM specification for read headers, where the first two letters (BX) are an arbitrary name (the common tag used for linked-read barcodes) and “Z” indicates the data type is any alphanumeric value, including spaces. Demultiplexing both removes barcodes from the sequences, storing the barcode in the sequence header, and splits bulk sequence data into separate paired-end FASTQ files per individual according to a user-provided sample-barcode schema.

### 3.2 Quality control

The Harpy quality control module includes the removal of sequencing adapters, trimming low-quality regions and removing poly-G tails, which is accomplished with fastp (Chen *et al.* 2018). In addition to these default routines, the program supports optical deduplication, and a host of additional quality control methods.

Linked read datasets are subject to barcode sharing by unrelated molecules by chance (i.e. reads are unlikely to have originated from the same molecule), making it necessary to deconvolute data. This deconvolution can optionally be accomplished during sequence quality-assessment via QuickDeconvolution (Faure and Lavenier 2022), which uses a Kmer-based strategy to perform the deconvolution (Supplementary Material S1).

### 3.3 Sequence alignment

In the Harpy *align* module, there are three options to map reads to a reference genome: the BWA-MEM aligner (Li and Durbin 2009), the EMA aligner (Shajii *et al.* 2018), which uses the BWA algorithm to perform linked-read-barcode-aware (hereafter “barcode-aware”) sequence alignment, and the recent strobemer-based alignment method strobealign (Sahlin 2022). Unlike EMA, the BWA-MEM and strobealign methods are not barcode-aware (i.e. do not take the linked-read information into account when mapping reads), but the Harpy *align* workflow retains the BX: Z tag information from the sequence header into the alignment record. The *align* methods also use linked-read barcodes to improve marking duplicates.

**Figure 3.**
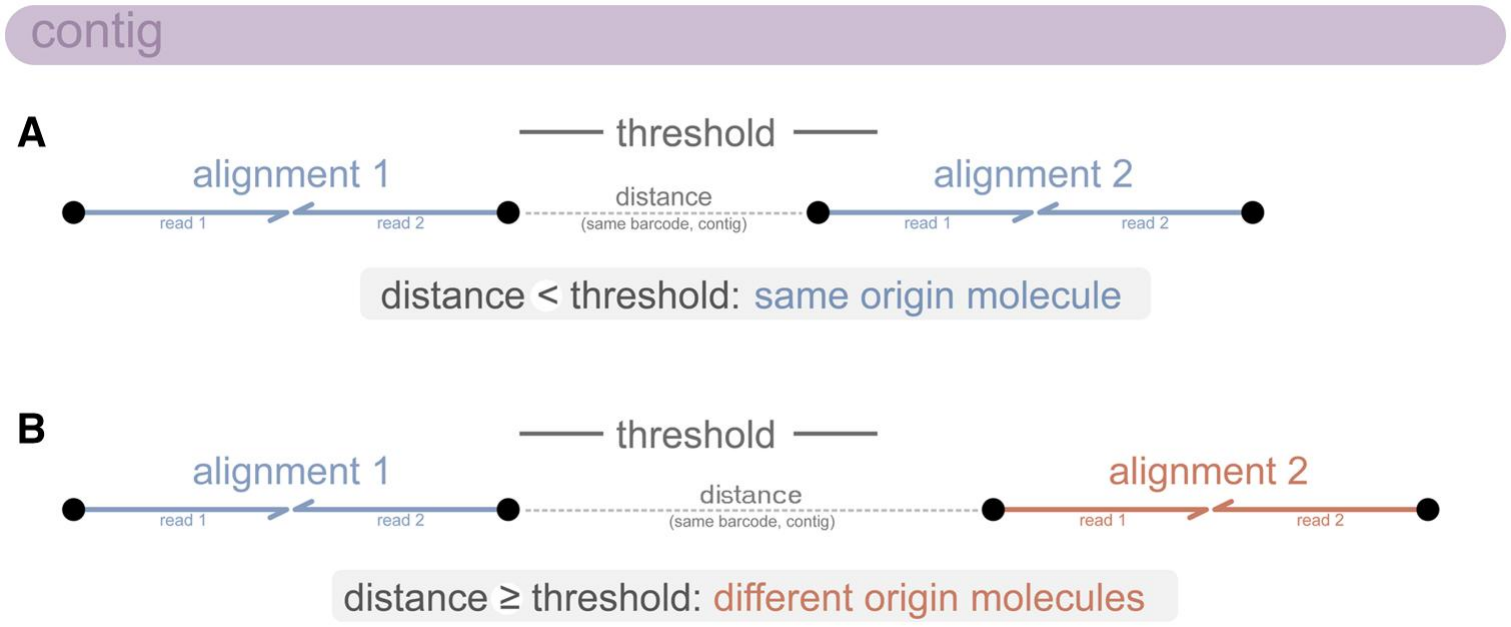
Distance-based deconvolution of linked-read barcodes shared across sequence alignments. (A) Two alignments with the same linked-read barcode (BX) align onto a single contig within a specified distance threshold, and both are assigned to the same original molecule. (B) Two alignments with the same linked-read barcode align onto a single contig greater than a specified distance threshold, and each alignment is assigned to a different molecule.

The EMA aligner uses internal heuristics to deconvolve linked-read barcodes and assign alignments to a unique molecule of origin. However, BWA-MEM and strobealign do not provide deconvolution, so Harpy optionally uses a custom distance-based algorithm to assign alignments to unique molecules of origin (Supplementary Material S1). Alignments are assigned to the same molecule if they have the same linked-read barcode and align to the same contig within a specified base-pair distance (e.g. 100 kbp). Alignments with the same barcode aligning to different contigs or appearing along the same contig with a distance greater than a specified threshold will be assigned to different molecules (Fig. 3).

### 3.4 SNP variant calling

In the Harpy *snp* workflow, variant calling (i.e. SNPs and small indels) is performed using bcftools mpileup (Danecek *et al.* 2021) or freebayes (Garrison and Marth 2012). These variant callers do not incorporate linked-read information, however, at the time of this writing there do not yet exist linked-read aware SNP variant callers for non-tumor data.

### 3.5 Haplotype phasing

The Harpy *phase* workflow uses HapCUT2 (Edge *et al.* 2017) to leverage linked-read information to phase SNPs into haplotype blocks. Phasing is performed on all samples at once, which tends to be more performant for lower-depth data. The Harpy NAIBR workflow (described below) can use the phased VCF file output from HapCut2 as input to phase sequence alignments (bam files) as input for structural variant detection.

### 3.6 Large structural variant calling

Harpy provides routines to call structural variants (e.g. inversions, large deletions, and duplications) using LEVIATHAN (Morisse *et al.* 2021) and NAIBR (Elyanow *et al.* 2018). LEVIATHAN requires linked-read sequence alignments with supplementary alignments, whereas NAIBR requires haplotype-phased alignments as input, which are not produced by the included sequence aligners. Despite linked-read data already containing phase information, there are no existing methods as of this writing to generate phased alignment data directly during haplotagging sequence alignment. Therefore, the Harpy NAIBR workflow uses WhatsHap (Martin *et al.* 2023) to phase sequence alignments using a haplotype-phased Variant Call Format (VCF) file, such as the one created by HapCut2 in the Harpy *phase* module. Structural variant calling can be performed per-individual or by optionally pooling samples into populations (analogous to pool-seq), which can be more performant on low-depth data.

### 3.7 Genotype imputation

Harpy uses STITCH (Davies *et al.* 2016) to impute missing genotypes using genotype likelihoods that are reported in the VCF outputs of either bcftools mpileup or freebayes. The STITCH software is unique in that it uses linked-read information to inform imputation and does not require a reference panel. Imputation performance can vary greatly with different parameters, and the STITCH documentation advises exploring the parameter space by testing the outcome of a few significant parameters. To explore this parameter space, Harpy accepts a plain-text table of input parameter values for each iteration of the optimization process.

## 4 Discussion

We developed the user-friendly Harpy linked-read analysis software for Linux-based systems to process raw haplotagging FASTQ files through configurable modules that will result in SNP variant calls, haplotype blocks of phased variants, structural variant calls, or genome assemblies, depending on project goals. Users interact with Harpy as command-line software (Supplementary Material S2), while Harpy preprocesses and validates inputs and configurations to launch Snakemake for dispatching complex workflows. This design all but removes the burden of a user needing to learn a complex workflow language and instead focus their investment on obtaining reproducible results. Rather than requiring all configurations for all workflows at runtime, Harpy is designed to be modular and provides users both with the flexibility to explore the parameter space within any given workflow, while also making the individual components of an end-to-end solution digestible to new users. Complementing these design decisions, Harpy also provides rich reports with summary information, quality control statistics, and visualizations for the various modules, further facilitating use and adoption.

To encourage linked-read use in research as a whole, as of version 2, Harpy supports multiple linked-read technologies, in addition to advocating for the adoption of a standardized linked-read FASTQ data format that stores the linked-read barcode in the BX: Z tag (regardless of technology-specific barcode format), along with a VX: i tag that records the barcode validation (0 for invalid, 1 for valid). This SAM-compliant format retains barcode data, remains technology-agnostic, and provides software developers with a simple and consistent barcode format to build software around without having to account for all possible barcode style variations. The release of Harpy version 2 also supports processing standard whole genome sequence data by disabling the workflow components tailored for linked-read data.

## Supplementary Material

vbaf133_Supplementary_Data

## Data Availability

Harpy is a free and open-source software distributed under the GPL-3 license. The software, extensive documentation, and test data can be accessed at https://github.com/pdimens/harpy.
