# Supplementary material for: Harpy: a pipeline for processing haplotagging linked-read data
Source: Bioinform Adv. 2025 Jun 5;5(1):vbaf133. doi: 10.1093/bioadv/vbaf133 (PMC12198493; doi:10.1093/bioadv/vbaf133)
Supplement: vbaf133_Supplementary_Data [file vbaf133_supplementary_data.zip › SupplementaryMatrial_I.pdf]

Supplementary Material I

Deconvolution

Deconvolution describes identifying linked-read barcodes shared by fragments from different molecules, such that downstream applications are not confounded by incorrectly associating them as originating from a single DNA molecule. Deconvolution during the quality control stage of sequence data uses a Kmer-based approach to remedy this. If intending to assemble a genome, deconvolution at this stage would be necessary.

An alternative deconvolution method is to use alignment-based distance thresholds. Alignments are assigned to the same molecule if they have the same linked-read barcode and align to the same contig within a specified base-pair distance (e.g. 100 kbp). Alignments with the same barcode aligning to different contigs or appearing along the same contig with a distance greater than a specified threshold will be assigned to different molecules. However, a distance-based approach may worsen identifying large structural variants because linked alignments may be farther apart than the distance threshold due to large inversions or on different chromosomes due to translocations.

Genome Assembly

The unique molecular linkage information present in linked-read sequences data lends itself to improve genome assembly performance (Hulse-Kemp *et al.*, 2018; Ott *et al.*, 2018). This also translates well into metagenomic assemblies, where the linked-read information between sequences can be used by assembly algorithms to more confidently combine sequences into contigs within a single sample, species, or operational taxonomic unit (Zhang *et al.*, 2020). There exist methods with which to leverage the linked-read information to generate assemblies from the short-read sequences themselves. For a single sample, Harpy employs cloudSPAdes (Tolstoganov *et al.*, 2019) to generate an initial assembly, followed by improving the assembly using tigmint (Jackman *et al.*, 2018) to correct missassemblies, and ARCS (Yeo *et al.*, 2018) and LINKS (Warren *et al.*, 2015) to scaffold contigs. For a metagenomic assembly, Harpy first employs cloudSPAdes for the initial contig-level assembly, followed by athena (Bishara *et al.*, 2018) for an improved scaffold-level assembly. Both assembly workflows output assembly metrics via QUAST (Gurevich *et al.*, 2013) and BUSCO (Simão *et al.*, 2015).

Data Simulation

When possible, it is important to evaluate the utility of a tool or technology before investing in the up front financial and labor costs of adopting it. To evaluate the value of linked-read sequencing for a particular species or inference type prior to adoption, Harpy includes workflows to simulate raw linked-read data. First, Harpy employs simuG (Yue and Liti, 2019) for simulating variants (SNPs, indels, inversions, copy number variants, translocations) into an existing genome assembly. The workflow has the flexibility to simulate a diploid genome from a haploid assembly if run sequentially multiple times. Second, Harpy uses a modified version of

LRSIM (Luo *et al.*, 2017) to simulate linked-read sequences from an input genome assembly, such as the aforementioned simulated diploid genome. This workflow can be tailored to achieve a desired sequencing coverage and fragments per molecule. Collectively, these two workflows enable users to assess the value of haplotagging linked-read data on systems of interest prior to any financial investment in the technology.

Software Dependencies

An end-to-end workflow for linked-read data requires many dependencies, each with their own dependencies and installation nuances. To account for this, the base Harpy installation includes only the necessities of invoking Snakemake and performing file validations. Runtime dependencies for workflows are contained in versioned environment recipes that Snakemake downloads and installs at runtime. Alternatively, versioned environments are also provided in a Docker container that are used at runtime. The Docker container is versioned to match the Harpy version being used, guaranteeing consistent behavior for any given version of Harpy. Both software deployment approaches automatically install the environments or container, respectively, and the user is not expected to install any software manually beyond Harpy itself.

Bibliography

Bishara,A. *et al.* (2018) High-quality genome sequences of uncultured microbes by assembly of read clouds. *Nat. Biotechnol.*

Gurevich,A. *et al.* (2013) QUAST: quality assessment tool for genome assemblies. *Bioinformatics*, **29**, 1072–1075.

Hulse-Kemp,A.M. *et al.* (2018) Reference quality assembly of the 3.5-Gb genome of Capsicum annuum from a single linked-read library. *Hortic. Res.*, **5**, 4.

Jackman,S.D. *et al.* (2018) Tigmint: correcting assembly errors using linked reads from large molecules. *BMC Bioinformatics*, **19**, 393.

Luo,R. *et al.* (2017) LRSim: A Linked-Reads Simulator Generating Insights for Better Genome Partitioning. *Comput. Struct. Biotechnol. J.*, **15**, 478–484.

Ott,A. *et al.* (2018) Linked read technology for assembling large complex and polyploid genomes. *BMC Genomics*, **19**, 651.

Simão,F.A. *et al.* (2015) BUSCO: assessing genome assembly and annotation completeness with single-copy orthologs. *Bioinformatics*, **31**, 3210–3212.

Tolstoganov,I. *et al.* (2019) cloudSPAdes: assembly of synthetic long reads using de Bruijn graphs. *Bioinformatics*, **35**, i61–i70.

Warren,R.L. *et al.* (2015) LINKS: Scalable, alignment-free scaffolding of draft genomes with long reads. *Gigascience*, **4**, 35.

Yeo,S. *et al.* (2018) ARCS: scaffolding genome drafts with linked reads. *Bioinformatics*, **34**, 725–731.

Yue,J.-X. and Liti,G. (2019) simuG: a general-purpose genome simulator. *Bioinformatics*, **35**, 4442–4444.

Zhang,L. *et al.* (2020) A comprehensive investigation of metagenome assembly by linked-read sequencing. *Microbiome*, **8**, 156.
