# Supplementary material for: Harpy: a pipeline for processing haplotagging linked-read data
Source: Bioinform Adv. 2025 Jun 5;5(1):vbaf133. doi: 10.1093/bioadv/vbaf133 (PMC12198493; doi:10.1093/bioadv/vbaf133)
Supplement: vbaf133_Supplementary_Data [file vbaf133_supplementary_data.zip › SupplementaryMaterial_II.pdf]

Supplementary Material II

Harpy Workflow Example

These supplementary materials briefly describe a common linked-read data processing workflow when starting with FASTQ files and intending to:

- call SNPs
- call SVs
- impute genotypes
- phase SNPs

To demonstrate this, the code below first simulates a 1Mb genome with a single chromosome, then creates linked reads from it, then follows Harpy workflows to accomplish the tasks listed above. If intending to follow along with this document, you will need to install the software dependencies, which can be accomplished using conda or pixi. For brevity, the directions below are given to create a Pixi environment.

```
pixi init -c conda-forge -c bioconda .
```

```
pixi add harpy=1.16 mimick seqtk seqkit snakemake-minimal=8.30
```

Simulate linked-read data

First, we must create a randomized reference genome. To simplify this and avoid additional software dependencies, we can use basic BASH commands to randomize the ATCG nucleotides, then make sure the output FASTA file has no formatting issues by parsing it with seqtk seq. If using real data, then performing these simulations are not necessary.

```
mkdir -p data
echo ">contig_1" > data/genome.fasta.tmp
shuf -re A T C G | tr -d '\n' | head -c 1000000 >> data/genome.fasta.tmp
cat data/genome.fasta.tmp | seqtk seq > data/genome.fasta
rm data/genome.fasta.tmp
```

For the sake of completeness, it would be sensible to create a second haplotype to simulate a "diploid" genome. Here, we will create an inversion by reversing a 100kb section of the genome.

```
start=50000
end=150000

# Extract the part before the section being inverted
head -c $((start - 1)) data/genome.fasta > data/genome.hap2.fasta.tmp

# Extract, reverse, and append the target section
cut -c ${start}-${end} data/genome.fasta | rev >> data/genome.hap2.fasta.tmp

# Append the part after the inversion
```

```
tail -c +$((end + 1)) data/genome.fasta >> data/genome.hap2.fasta.tmp
cat data/genome.hap2.fasta.tmp | seqtk seq > data/genome.hap2.fasta && rm
data/genome.hap2.fasta.tmp
```

With a diploid genome created, we can simulate linked-reads from the two haplotypes using Mimick, a purpose-built software spawned from VISOR/XENIA and integrated into the forthcoming Harpy v2. This will randomly generate 96 6bp combinatorial barcodes. Doing it twice to create two "samples" with these characteristics:

- flat error rate of 0.0001 for all base pairs
- a coverage of 20X
- no simulated indels
- default random mutation rate (0.02)
- exactly one molecule per barcode, an unrealistic simplification for demonstration purposes

```
for i in {1..2}; do
      mimick --lr-type haplotagging -o data/sample_0${i}/sample_0${i}
      --error 0.0001 -q 2 --coverage 20 --indels 0 -n -1 6,96
      data/genome.fasta data/genome.hap2.fasta
done
```

Mimick outputs separate paired-end files per haplotype, so it will be necessary to merge them together using cat. However, Mimick does not guarantee proper pairing of paired-end reads, so we will use seqkit to make sure the reads are properly paired and remove unpaired reads.

```
for i in {1..2}; do

      cat data/sample_0${i}/sample_0${i}.hap_00{1..2}.R1.fq.gz >

            data/sample_0${i}.R1.fq.gz

      cat data/sample_0${i}/sample_0${i}.hap_00{1..2}.R2.fq.gz >

            data/sample_0${i}.R2.fq.gz

      # make sure the reads are properly paired

      seqkit pair --id-regexp '^(\S+)\/[12]' -1 data/sample_0${i}.R1.fq.gz

            -2 data/sample_0${i}.R2.fq.gz

      rm data/sample_0${i}.R{1..2}.fq.gz

      mv data/sample_0${i}.R1.paired.fq.gz data/sample_0${i}.R1.fq.gz

      mv data/sample_0${i}.R2.paired.fq.gz data/sample_0${i}.R2.fq.gz

done
```

Quality Checking FASTQ Files

Quality checking in genomics workflows includes adapter removal, quality trimming, length trimming, deduplication, etc. Since the simulated data does not feature adapters, we will not opt-in to those Harpy features, instead favoring a simple quality-trimming with the default parameters. The subsequent Harpy commands in this document will follow a familiar pattern of harpy <command> <subcommand, optional> <options> <inputs>

```
harpy qc data/sample*.fq.gz
```

Align to Reference

Once the samples have been quality-assessed, you can view their various reports/outputs in the QC/reports directory. If you are satisfied with the sequence data, then you can proceed to align the quality-checked reads to the reference without the inversion, genome.fasta. Harpy offers BWA-MEM, EMA, and strobealign aligners, and the example below features BWA. Despite BWA not being linked-read aware, the routines within Harpy will carry the linked-read barcode into the alignment records for downstream processing.

```
harpy align bwa -q 20 -d 9999999 -o Align -g data/genome.fasta QC
```

Harpy will generate various logs to Align/logs as well as interactive reports to Align/reports, including summary information across all samples and linked-read statistics based on alignment.

Call SNPs with the alignments

Once reads have been aligned, you can call SNPs using either mpileup or freebayes. Their performance is comparable, however freebayes can call SNPs on ploidy > 2, so mpileup is shown below. The default settings will parallelize SNP calling over genomic intervals and merge the results.

```
harpy snp mpileup -o SNP -g data/genome.fasta Align/
```

You can view the SNP calling report that will be present in SNP/reports/. The routines in Harpy will generate a file of "raw" SNP/indel calls, along with one where indels have been "normalized", sometimes known as left-aligned.

Call SVs with the alignments

Calling structural variants such as inversions or duplications can be done using either LEVIATHAN or NAIBR. The latter requires phased alignment data as input (or a phased VCF to phase the alignments), however NAIBR tends to perform better than LEVIATHAN. As a simple demonstration, we are using LEVIATHAN (via harpy sv leviathan) to call SVs. The modifications below specify that at least one barcode is needed to validate an SV candidate (-b 1), and we are supplying an extra argument (-x "--largeRate 90) to LEVIATHAN that Harpy does not expose at the command line. This extra command lowers the stringency of candidate SV detection and removal by filtering.

```
harpy sv leviathan -x "--largeRate 90" -o SV -b 1 -g data/genome.fasta Align/
```

You can view the SV calling report present in SV/reports. As a convenience, the Harpy routines will also aggregate the putative SV's by type into their own files. Regrettably, the simulated data likely won't identify any structural variants.

Phase the SNPs

If you were interested in phasing SNPs into haplotypes for analysis or calling SVs via NAIBR, you would use the harpy phase command. Below is a simple invocation of it, while also calling -g to include a reference genome required to use indels for phasing (in addition to SNPs).

```
harpy phase -g data/genome.fasta -v SNP/variants.normalized.bcf Align
```

You can view the phasing report in Phase/reports, which describes various metrics relating to phasing performance, such as molecule N50, haplotype size distributions, etc.

Impute Genotypes

To impute missing genotypes or get hard genotype calls from genotype likelihoods (e.g. low-coverage data), you would consider imputation. Unlike other Harpy commands, imputation requires a parameter file to explore the parameter space. Harpy includes a command to create a template parameter file for users to modify.

```
harpy imputeparams -o stitch.params
```

Once that file has been created and modified to include parameters appropriate for the study system, it will be used in the harpy impute command. The parameter file allows Harpy to

parallelize imputation over contigs and parameter sets, resulting in a better usage of system resources for what could be a time-consuming process.

```
harpy impute -p stitch.params -v SNP/variants.normalized.bcf Align
```

The resulting directory structure is more complicated than previous examples due to the hierarchical nature of the results (i.e. contigs and parameter sets). Inside Impute will be a folder for each parameter set, which can be considered independent analyses. Each will have its own logs and reports.
